# A Rare Case of Prurigo Pigmentosa in Iranian Sibling Couple

**DOI:** 10.1002/ccr3.70121

**Published:** 2025-01-16

**Authors:** Saman Al‐Zahawi, Fatemeh Saberi, Vahidesadat Azhari, Kambiz Kamyab, Kamran Balighi

**Affiliations:** ^1^ Department of Dermatology Razi Hospital, Tehran University of Medical Sciences (TUMS) Tehran Iran; ^2^ Autoimmune Bullous Diseases Research Center Razi Hospital, Tehran University of Medical Sciences Tehran Iran; ^3^ Department of Dermatopathology Razi Hospital, Tehran University of Medical Sciences (TUMS) Tehran Iran

**Keywords:** HLA, human leukocyte antigen, ketogenic diet, Nagashima disease, PP: Minocycline: Azithromycin, prurigo pigmentosa

## Abstract

Prurigo Pigmentosa is a rare inflammatory skin disease of unknown origin, characterized by pruritic, erythematous papules on the chest, back, neck, and anterior abdomen. The eruption resolves with reticular hyperpigmentation that cosmetically affects the patient's quality of life. Previous reports highlighted the role of the Ketogenic diet in triggering the disease in young female patients, however, no study reported the occurrence of Prurigo Pigmentosa in siblings of one family, unrelated to a ketogenic diet. Here, we report two sisters diagnosed with Prurigo Pigmentosa without undergoing a Ketogenic diet.

## Graphical abstract

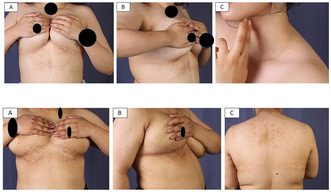

**Summary**

- The diagnosis of Prurigo Pigmentosa in two siblings may support the genetic basis of this dermatological condition and promote further study to find the specific HLA allele responsible for its occurrence.

## 1 Introduction

Nagashima observed the cyclic occurrence of pruritic papular lesions that coalesce to form more reticulated lesions on the chest, back, and neck of Japanese female patients undergoing Ketogenic diet in 1971, leaving hyperpigmentation on resolution [1]. Initial studies reported the typical occurrence of such lesions in young females of East Asian ethnicity, but subsequent studies reported Prurigo Pigmentosa (PP) in Caucasian, Black, and Middle Eastern countries [2, 3, 4]. Cases of PP as young as 7 years old and as old as 61 years old have been reported [2]. Although the etiology of PP is unknown, multiple factors are implicated as triggering events in its development, these include fasting following Ramadan in Middle Eastern countries, pregnancy, infection with SARS‐CoV2, COVID‐19 vaccination, Hormonal changes, Ketoacidosis and Ketogenic diet [5].

The diagnosis of PP is clinical; however, histopathology may be needed to rule out other similar conditions. Histopathological findings are not specific and change with the stage of the disease, whether it is early erythematous papules or late reticular hyperpigmentation. The main clinical differential diagnosis includes confluent and reticulated papillomatosis, darier disease, and reticulated and erythematous mucinosis. Confluent and reticulated papillomatosis unlike PP is not preceded by the erythematous papules. Darier disease tends to involve the seborrheic area including the scalp, face, and upper chest with a positive family history and possible nail changes. Reticulated and erythematous is not itchy and histologically abundant mucin is seen.

Treatment is directed initially against the inflammation, then the less responsive residual hyperpigmentation is treated by modalities like laser, phototherapy, and chemical peels [5]. Minocycline is a well‐established effective treatment for Prurigo Pigmentosa in an initial high dose of 100 mg twice daily, then a lower maintenance dose until complete resolution of the inflammation [6].

## 2 Case History/Examination

An 18‐year‐old female patient visited the Primary Center for Dermatological Diseases/Razi Dermatology Hospital/Iran with her sister for similar pruritic skin lesions on her chest and back. The elder sister was a 30‐year‐old lactating woman who developed the skin eruption just 1 month after giving birth, she lived far away from the younger sister, while the younger sister had the skin eruption for more than 1 year with periodic resolution and flare‐ups. Both patients denied any sort of dieting or fasting, with no significant past medical diseases.

**FIGURE 1:**
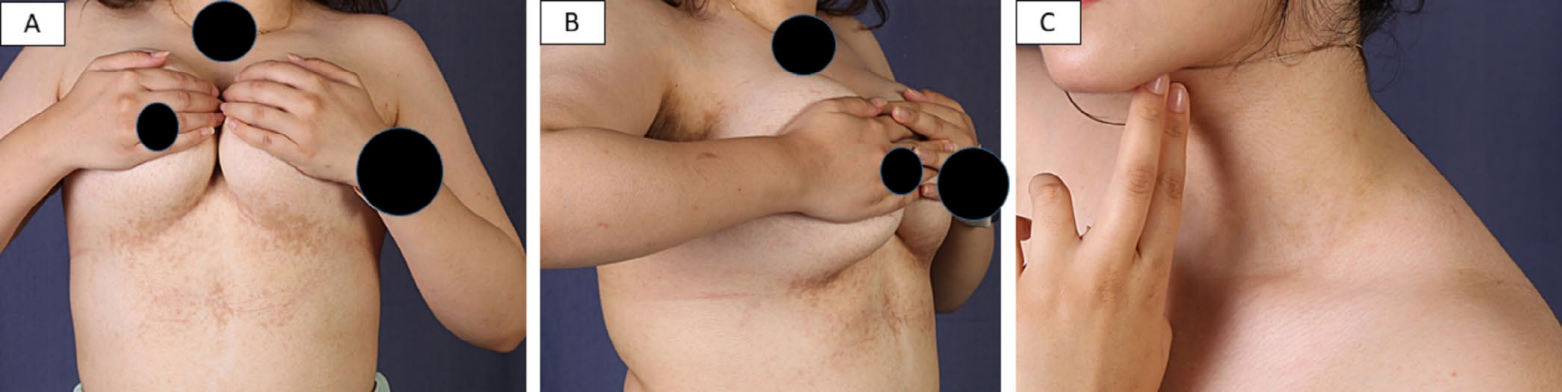
The younger sibling presented with reticular hyperpigmentation of the submammary region (A), lateral view of the submammary region (B), and very mild hyperpigmentation of the neck (C).

**FIGURE 2:**
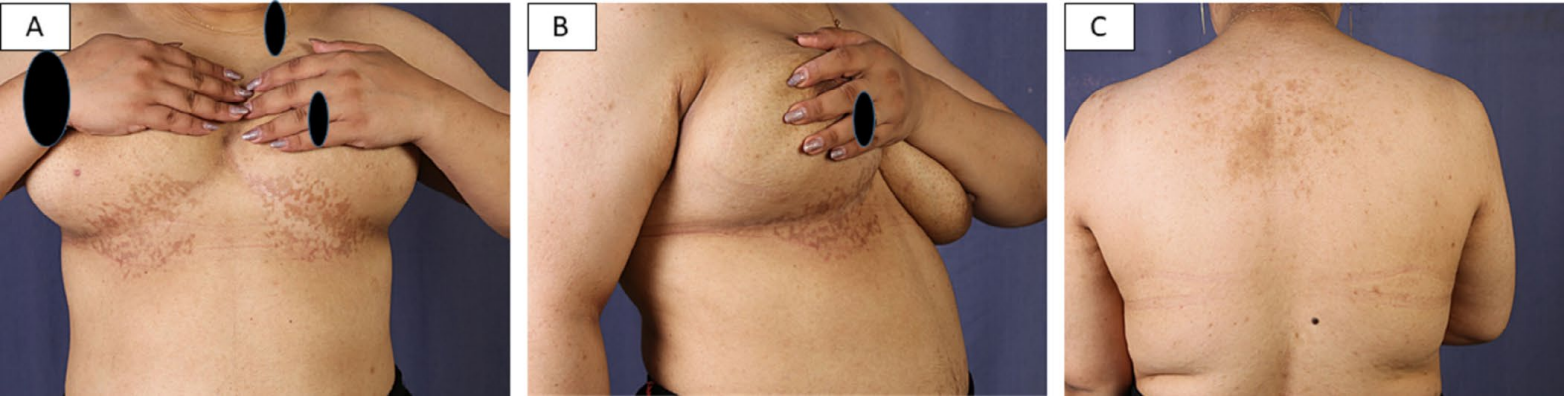
The elder sibling presented with reticular hyperpigmentation of the submammary region (A), few erythematous papules on the lower border of the reticulated patch on the right side (B), and reticular hyperpigmentation of the back (C).

On exam: the younger sibling showed reticulated macular hyperpigmentation on the chest and neck (Figure 1A–C), while the elder sibling showed few erythematous papules at the border of the reticulated patch of the chest, in addition to reticulated hyperpigmentation of the back (Figure 2A–C).

### 2.1 Differential Diagnosis, Investigations and Treatment

**FIGURE 3:**
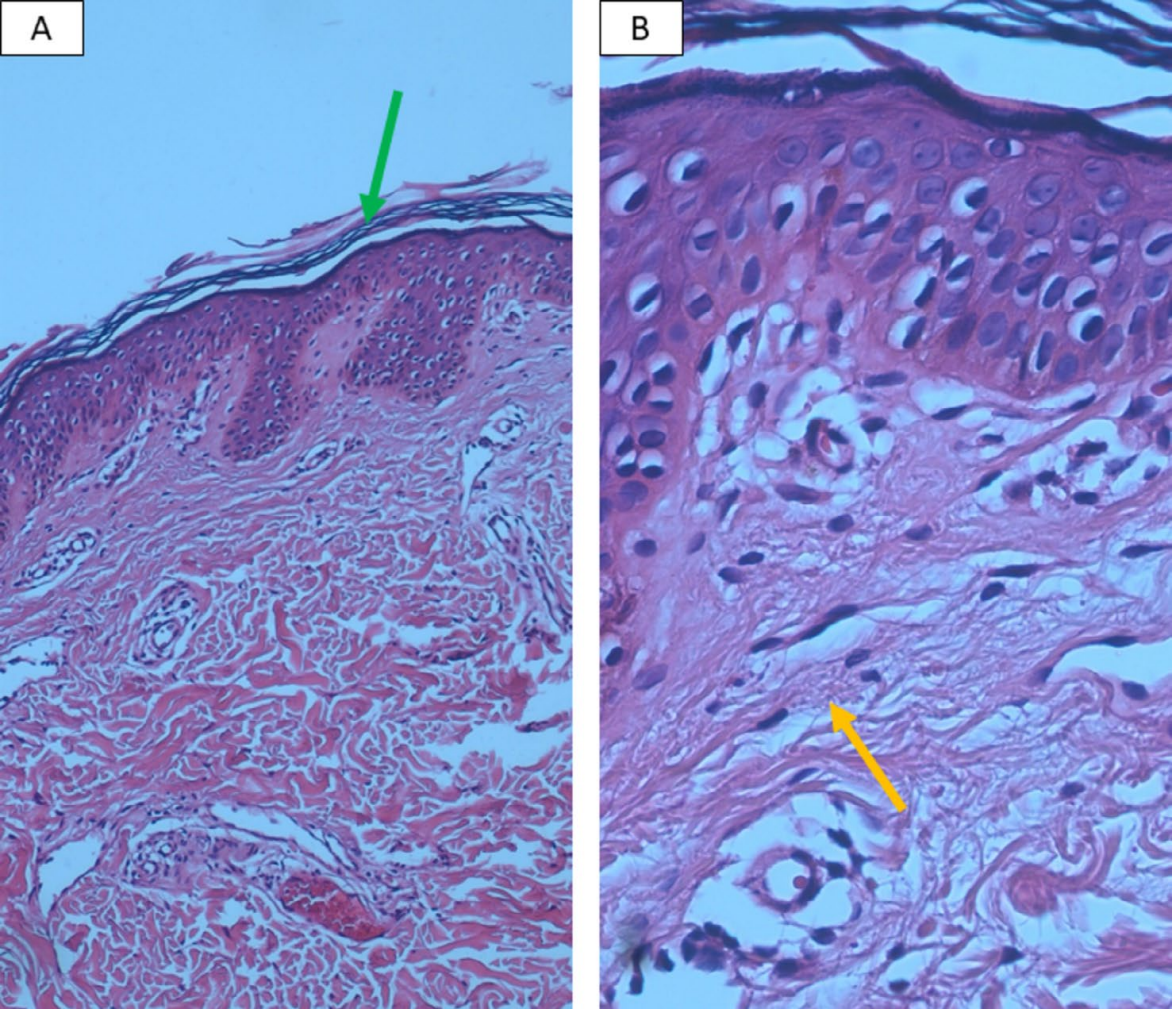
Punch biopsy from the reticulated hyperpigmented patch of the younger sibling revealed nonspecific changes with basket weave orthokeratotic cornified layer (green arrow), elongation of the rete ridges (A), and superficial dermal perivascular infiltrates of lymphocytes with very few melanophages (yellow arrow) (B).

**FIGURE 4:**
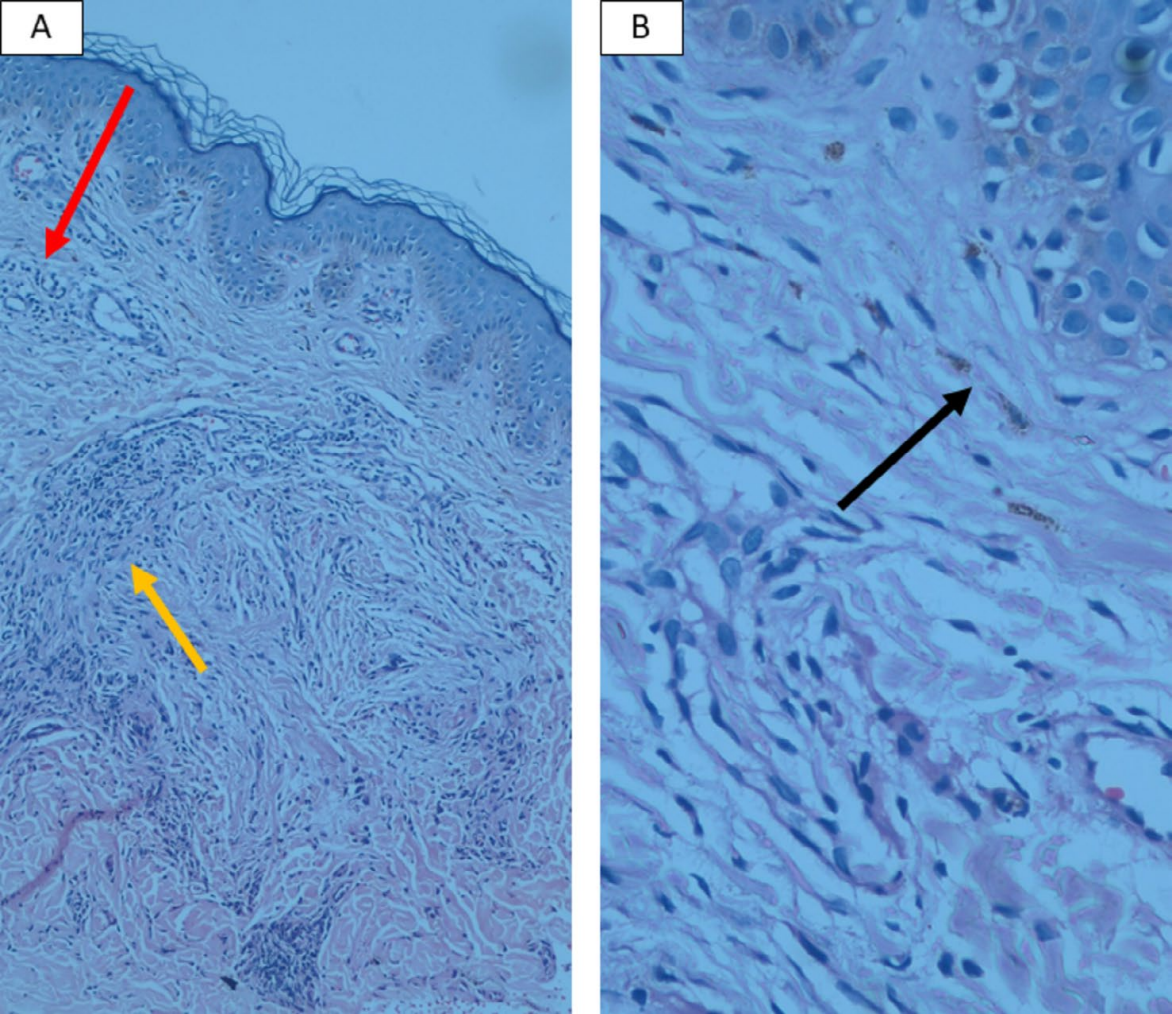
Punch biopsy from the erythematous papules of the elder sibling showed basket weave hyperkeratosis, slight acanthosis with elongation of rete ridges (A), dermal melanophages (black arrow) and perivascular infiltrates of lymphohistiocytes were observed (B) (red arrow), also, several foreign body type multinucleated giant cells are seen in the dermis with possible overlapping perifolliculitis (yellow arrow).

Metabolic panels were normal for glucose, ketones, insulin, and HbA1c in both patients. Punch biopsy from the reticulated hyperpigmented patch of the younger sibling revealed basket weave orthokeratotic cornified layer, elongation of the rete ridges, and superficial dermal perivascular infiltrates of lymphocytes with very few melanophages (Figure 3A,B). Also, Punch biopsy from the erythematous papules of the elder sibling showed basket weave hyperkeratosis, slight acanthosis with elongation of rete ridges, dermal melanophages, and perivascular infiltrates of lymphohistiocytes (Figure 4A,B). Diagnosis of Prurigo Pigmentosa was based upon the typical clinical presentation and the compatible pathological findings. The younger sibling was treated with minocycline 50 mg daily with subsequent follow‐up scheduling 1 month later and the elder sibling was treated with Azithromycin 250 mg daily as she was lactating and minocycline was not appropriate for her.

### 2.2 Outcome and Follow Up

**FIGURE 5:**
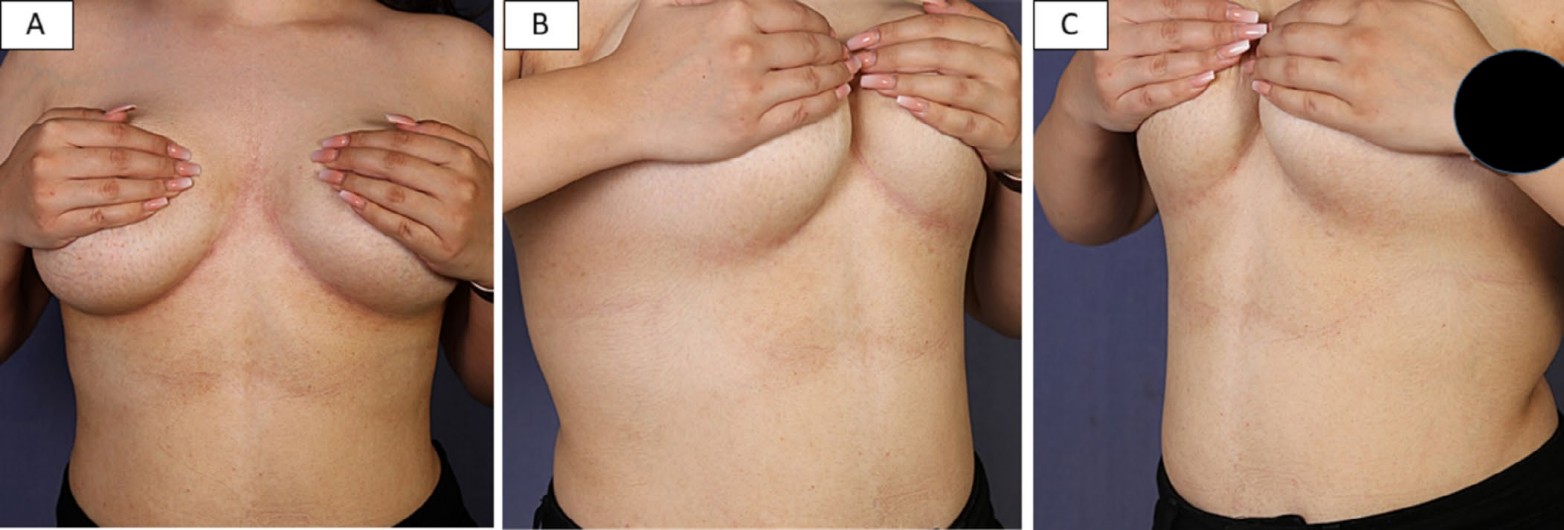
The younger sibling with acceptable clearance of the lesions after 1 month from minocycline therapy and whitening cream (A–C).

**FIGURE 6:**
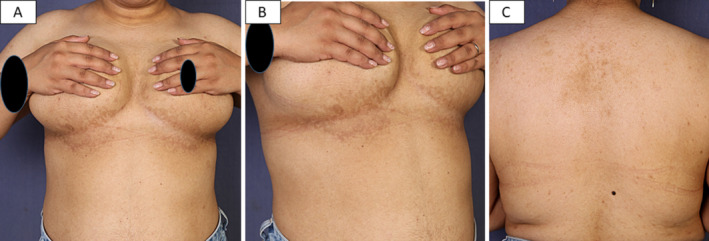
The elder sibling with slight improvement after 1 month from Azithromycin therapy and whitening cream in previuos sites, anterior chest (A), lateral side of the chest (B) and back (C).

The younger sister who received minocycline 50 mg showed a better response than the elder sister who received azithromycin after 1 month of therapy (Figures 5A–C and 6A–C). Whitening creams also was prescribed for the remnant hyperpigmentation.

## 3 Discussion

PP is a rare inflammatory skin disease with multiple well‐defined triggering factors and unknown etiology. Exclusion of carbohydrates in diet and ketosis are the main two triggering factors in the development of PP [7]. it has been suggested that the perivascular accumulation of ketones in patients undergoing a ketogenic diet will trigger a perivascular neutrophilic infiltration and subsequent skin lesions [8]. It is well established that not all individuals with ketosis experience such skin lesions, and it seems that certain genetic predisposition paves the way for the effect of ketones or other triggering factors to be exerted.

Initial clustering of cases in Japan, reporting of PP in monozygotic twins, PP in siblings, and segmental PP in some individuals highlight the importance of genetic basis in the development of PP and prompt further studies to define the specific HLA that plays a role in the pathophysiology of PP. Houriet et al. described the occurrence of PP in monozygotic twin females [9]. Also, A Danish sibling females were reported for having PP after undergoing a strict ketogenic diet [10]. Our case is similar to the Danish sibling in that it is familial, but unlike the Danish sibling, there was no evidence of a defined triggering factor in the younger sibling, while the elder sibling had the lesions after giving birth and 1 month after lactation. We postulate that hormonal changes, specifically lactation in the elder sibling after pregnancy triggered the PP; however, we didn't find a well‐defined triggering event in the younger sibling except for the occasional exacerbation of her lesions during menstrual cycles. The only way to relate the exacerbation of the skin lesions in the younger sibling during menstruation was the theory of catamenial hyperglycemia, which postulates the occurrence of hyperglycemia and subsequent ketoacidosis during the menstrual cycle in the diabetic patient, but she was not diabetic and this theory didn't explain the worsening of her lesions [11]. Overall, we postulate that hormonal changes in our case are the triggering factor for PP development with a strong genetic predisposition. Families in Middle Eastern countries are large, and inquiries about other family members with similar skin lesions and future findings of more than two siblings with similar lesions will further support the notion of specific HLA in the pathophysiology of PP. Another supportive evidence for the genetic basis of PP is the previous report of segmental PP in the left side of the chest in a 13‐year‐old female patient [12].

In patients with well‐defined triggering factors, the first step is to remove the triggering factor such as inserting carbohydrates into the daily diet, avoiding fasting, or stopping lactation in lactating females.

Spontaneous improvement of PP was reported after 1 month from stopping lactation [3], in our case the mother refused to stop lactation and asked for choices other than cessation of lactation. If the above measures were not effective at controlling the lesions, then medications that decrease neutrophilic infiltration could be used. These medications include minocycline, dapsone, and macrolides [5]. The younger sibling was given minocycline 50 mg as maintenance therapy and recommended to have a follow‐up visit to stop the medication and plan to treat the reticular hyperpigmentation. The elder sibling was given Azithromycin 250 mg daily as she was lactating and minocycline was not suitable for her. A comparable study showed no preference for the two antibiotics in achieving improvement within 4 weeks [13], but our study revealed that minocycline has more effect in clearing the lesions in PP than azithromycin. Treatment of reticular hyperpigmentation is difficult, even though complete spontaneous resolution after 3 months has been reported [3]. Successful measures to treat hyperpigmentation include laser therapy, chemical peels, microneedling, and topical agents like retinoids [5].

## 4 Conclusion

Overall, the diagnosis of Prurigo Pigmentosa in two siblings without undergoing a ketogenic diet supports a genetic basis for PP and further confirms the different triggering factors other than ketoacidosis. Future genetic studies may find a specific HLA related to the development of PP.

## Author Contributions

**Saman Al‐Zahawi:** data curation, writing – original draft, writing – review and editing. **Fatemeh Saberi:** writing – review and editing. **Vahidesadat Azhari:** data curation, visualization. **Kambiz Kamyab:** data curation, visualization. **Kamran Balighi:** conceptualization, supervision.

## Consent

Written informed consent was obtained from the patient to publish this report in accordance with the journal's patient consent policy.

## Conflicts of Interest

The authors declare no conflicts of interest.

## Data Availability

The authors elect to share data per request.
